# Selective ensemble method for anomaly detection based on parallel learning

**DOI:** 10.1038/s41598-024-51849-3

**Published:** 2024-01-16

**Authors:** Yansong Liu, Li Zhu, Lei Ding, Zifeng Huang, He Sui, Shuang Wang, Yuedong Song

**Affiliations:** 1 School of Software Engineering, Xi’an Jiao Tong University, Xi’an, China; 2 School of Intelligent Engineering, Shandong Management University, Jinan, China; 3 School of Cyberspace Security, Guangzhou University, Guangzhou, China; 4 School of Electronics and Communication Engineering, Guangzhou University, Guangzhou, 510006 China; 5 College of Aeronautical Engineering, Civil Aviation University of China, Tianjin, 300300 China; 6 Information Security Evaluation Center of Civil Aviation, Civil Aviation University of China, Tianjin, 300300 China; 7 Shanghai Hua Xun Network Information System Co., Ltd, Shanghai, 200135 China

**Keywords:** Mathematics and computing, Applied mathematics, Information technology

## Abstract

Anomaly detection is a highly important task in the field of data analysis. Traditional anomaly detection approaches often strongly depend on data size, structure and features, while introducing the idea of ensemble into anomaly detection can greatly improve the generalization ability. Ensemble-based anomaly detection methods still face some challenges, however, such as data imbalance, time and space demand and the selection of base detectors. To this end, we propose a selective ensemble method for anomaly detection based on parallel learning (SEAD-PL). First, a differentiated stratified sampling method is designed to alleviate the problem of data imbalance. Then, a distributed parallel training frame is built to address the problem of excessive time and space consumption for base detector training. Finally, a clustering-based ensemble selection strategy is introduced to balance the accuracy and diversity of base detectors. Experiments are performed on six datasets, which demonstrate that the proposed method has obvious advantages over four selected methods.

## Introduction

Anomaly detection is a crucial task in the field of data analysis, making it a popular research topic in machine learning. Upon the occurrence of some kind of malicious attack or information leakage in a complex network, obvious anomalies will be produced by the network traffic, the timely detection of which is essential to guarantee network security.

Anomaly detection can be regarded as a special kind of binary classification problem consisting of anomaly classes and normal classes. Meanwhile, traditional classification algorithms are not directly applicable to anomaly detection because of its special nature. Anomaly detection became an independent research topic when its general definition was proposed by Hawkins^1^. Building on his research, density-based and distance-based approaches were gradually developed^2^. The former assume that normal samples in the dataset are distributed in dense neighborhoods, while anormal samples are distributed in sparse neighborhoods, i.e., they are far away from their neighbors^3^. Such methods do not rely on any assumptions about data distribution; however, they show poor performance in case of local anomalies. On the other hand, distance-based methods^4^ assume that normal samples in a dataset are relatively close to those neighbors, while the distance of anormal samples from their neighbors is comparatively large. These methods are data-driven approaches that do not need to assume the data distribution in advance, whereas they are inferior at detecting anomalous clusters.

Traditional anomaly detection algorithms are often designed specifically for a certain domain, with strong dependence on data size, structure and features, giving them limited applicability in the detection of large data amounts and multiple data from several domains with large generalization errors. As an important method in the field of machine learning, ensemble learning has been shown to provide significant improvement to the generalization ability of algorithms as early as in the classification and clustering tasks^5,6^. Introducing the idea of ensemble into anomaly detection reduces the dependence of traditional methods on a specific dataset and a single model, which can greatly enhance the generalization ability of anomaly detection and effectively increase the utility of the algorithm.

Zhou^7^ asserted that an effective ensemble method should take into account the accuracy and diversity of each base classifier. To meet the diversity requirement, Liu et al.^8^ proposed a segregation-based anomaly detection algorithm that selects binary trees with randomness as base classifiers, carried out sample partitioning by the random selection of attributes of different subsample sets, and finally used the path length of the tree as an important reference criterion. Latecki et al.^9^ integrated the results of basic detectors using different feature sets, where each detector employs a subset of features by random selection. This method demonstrated good performance on large, high-dimensional or noisy datasets. Nguyen et al.^10^, Kriegel et al.^11^ and Schubert et al.^12^ also proposed various ensemble anomaly detection algorithms.

In summary, the idea of ensemble is to simply train the base detectors first and then combine them using a certain strategy. The obtained effect mainly depends on the training results of base detectors and the setting of ensemble strategy, while the efficiency and cost of ensemble detection must also be taken into account. To this end, ensemble anomaly detection still faces the following challenges:

- Due to the need to balance effectiveness and efficiency, the base detectors should not be overly complex; however, the actual data size is large and extremely imbalanced. Thus, simple base detectors lack the ability to deal with these data, which leads to low performance and impairs the detection effect.
- Ensemble methods need more time and space compared to single detection models, especially in the training phase; a large amount of data is used to train multiple base detectors, which is a challenge in terms of computing power and storage. Therefore, it is important to efficiently train base detectors with lower time and space requirements.
- Using the ensemble for all base detectors does not consistently improve the detection performance, and the selection of better-performing base detectors to participate in the ensemble will not achieve the desired results either. As such, the diversity of base detectors is an important factor to be considered, and the means to find a balance between accuracy and diversity has become an urgent task for base detector selection.

In order to address the above challenges, we propose a selective ensemble method for anomaly detection based on parallel learning (SEAD-PL). The main characteristics of this method are as follows:

1. For the performance enhancement of base detectors, a differentiated stratified sampling method is designed. A subset of large class samples with the same number of small class samples is randomly extracted and then composed with small class samples to form multiple new balanced subsets for base classifier training, so as to alleviate the problem of unbalanced training data.
2. To improve the training efficiency of base detectors, a distributed parallel training model is proposed. Based on the MapReduce framework, the training data are distributed to each node and the edge processing capability of the distributed nodes is fully utilized for the training of base detectors.
3. Regarding base detector selection, a clustering-based strategy is proposed. The measure of inconsistency is taken to characterize the differences between base detectors, which are then fuzzy clustered to determine the ensemble scale. Subsequently, the best-performing base detectors in each cluster are selected to participate in the ensemble model.

The main content of this paper is organized as follows. In “Related works” section, we introduce the related works on imbalanced data anomaly detection, discuss the base detector training model in ensemble learning, and explain the base detector selection strategies for ensemble. In “The SEAD-PL approach” section, we describe the proposed SEAD-PL approach in detail. Experimental studies and the discussion of results are conducted in “Experimental methodology” section. Finally, the conclusions and future outlook are expanded in “Conclusions” section.

## Related works

### Anomaly detection for imbalanced data

Class imbalance is mainly reflected in the following three aspects^13^: (1) great differences in data volume; (2) imbalanced sample distribution; (3) samples overlapping in the feature space. As a result, data with little anomaly are always buried in a sea of normal data^14^. Therefore, it is an urgent task to overcome class imbalance and to obtain high detection accuracy for anomaly detection. In general, solutions are attempted using three main strategies: data processing, feature extraction, and algorithm optimization. Obviously, data processing is easier as there is no requirement to understand the loss function, such as in resampling^15^, including oversampling and undersampling.

The most classic oversampling method is random SMOTE, which generates samples with linear interpolation. Despite its effectiveness, recent studies have reported that noisy data are usually responsible for its degeneration. Therefore, several methods based on SMOTE have been developed, such as ADASYN^16^, MWMOTE^17^, NI-MWMOTE^18^, MOKAS^19^, and PAIO^20^. Oversampling needs sufficient information to generate high-quality data; however, it is hard to capture the distribution of minority samples, so as to generate low-quality samples deviating from the actual distribution. Thus, producing more diverse and higher-quality samples remains a critical task.

The simplest undersampling method performs random undersampling, which comprises the random removal of samples of the majority class until the expected distribution is reached. However, a large number of sample features are lost along the process, hence the accuracy of sample classification cannot be significantly improved. To solve this problem, scholars have proposed undersampling methods focusing on overlap elimination based on clustering or its variant, such as Edited Nearest Neighbors (ENN)^21^, Tomek Link (TL)^22^, and Neighborhood Cleaning Rule (NCL)^23^. A sensitivity-based undersampling method by clustering^24^ has also been introduced to undersample the majority instances, which also uses nearest neighbor search.

On issue is that the quality of generated samples is not guaranteed, while deleting the samples will lose some important information, both of which are unfavorable for the training of base classifier. Therefore, in this paper, we make full use of the original data and re-group the data for balancing in each group, so as to improve the training effect of the base classifier.

### Training and selection of base detector for ensemble learning 

The training mode of base detectors can be divided into two categories: parallel training and sequence training. Araya et al.^25^ proposed a parallel anomaly framework for the detection of anomalous energy consumption behaviors generated during the operation of a building, which implements the ensemble of three common anomaly detection algorithms. The common hyperspectral anomaly detection algorithms usually score anomalies by finding a single approximation kernel, a process that is susceptible to anomalous samples. To mitigate this problem, Merrill et al.^26^ proposed a kernel principal component analysis-based parallel anomaly ensemble algorithm for anomalous pixel point detection in hyperspectral images by integrating multiple models. Regarding sequence training, due to the problem of missing data labels in anomaly detection, there are relatively few sequences training-based anomaly ensemble algorithms. Rayana et al.^27^ presented an anomaly detection algorithm combining parallel ensemble and sequence ensemble, in which the parallel block is used to combine the results of multiple basic classifiers and the sequence block is used to successively eliminate the potentially anomalous samples from the original dataset and construct a better training data for anomaly evaluation. Obviously, the parallel method has higher efficiency and the training of base classifiers is less dependent on prior knowledge.

There are three main types of strategies for the selection of base classifiers for integrated models: ranking-based, clustering-based and optimization-based^28,29^. The main idea of ranking-based selection is that the performance of the base classifiers is ranked according to a certain algorithm, and the preferred selection is based on the ranking result^30^. The essence of clustering-based selection is to first use a clustering algorithm to classify the base classifiers into different clusters and then take the results of clustering to select base classifiers for the ensemble^31^. On the other hand, optimization-based selection methods usually assign selection weights to each base classifier, represent the weights as a vector, and obtain the weight vector through optimization, which is then selected according to a certain strategy^32,33^.

### Application of neural network and ensemble learning for detection

Ensemble learning has been widely used in some applications, such as image recognition and data analysis. Hong et al.^34^ employed multimodal data and proposed a novel face-pose estimation ensemble framework, which is based on feature extraction with improved convolutional neural networks (CNNs) and a multimodal mapping relationship with multitask learning. The authors also proposed a new pose recovery method using non-linear mapping with multi-layered deep neural network^35^, which is formulated on feature extraction with multimodal fusion and back-propagation deep learning. As a result, the recovery error was reduced by 20–25%. Yu et al.^36^ proposed a novel ranking model based on learning to rank the framework, in which visual features and click features are utilized simultaneously to obtain the ranking mode. Specifically, the proposed approach is based on large margin structured output learning, and the visual consistency is integrated with the click features through a hypergraph regularizer term. Furthermore, the authors also devised a Hierarchical Deep Word Embedding model by integrating sparse constraints and an improved RELU operator to address click feature prediction from visual features^37^. In particular, the integration of different types of features is difficult for multiview data, thus a novel approach by adopting multiview locality-sensitive sparse coding in the retrieving process was proposed to recover 3-D human poses from silhouettes^38^. This strategy incorporates a local similarity preserving term into the objective of sparse coding, which groups similar silhouettes to alleviate the instability of sparse codes.

Object detection is another important application of neural network and ensemble learning, and its task is similar to anomaly detection. However, the reliability and localization accuracy of weakly supervised object detection are insufficient. To address these two shortcomings, Qian et al.^39^ proposed a semantic segmentation guided pseudo-label mining model that uses a novel metric named class-specific object confidence score to mine high-quality instances. They further developed a pseudo soft label assignment strategy to assign a more precise soft label for each instance, where the soft label is determined by the spatial distance between each instance and its nearest pseudo ground-truth instance^40^. Li et al.^41^ presented a few-shot object detection method with confidence collaborative proposal filtration and tiny object constraint loss, which proved to be effective in object detection within optical remote sensing images (DIOR), tiny object detection for aerial images (AI-TOD), and high-resolution remote sensing detection (HRRSD) datasets. Focusing on oriented object detection, Qian et al.^42^ developed a unified transferring strategy to facilitate the transfer of bounding box regression loss from horizontal object detection to oriented object detection. Comparisons with other losses demonstrated that the proposed transferring strategy can achieve better performance.

## The SEAD-PL approach

### Motivation

Ensemble learning can improve learning performance by the addition of sub-learning machines. However, two general problems persist in the current ensemble learning algorithms: poor generalization ability and learning efficiency.

For the generalization ability of ensemble learning, the universal applicability of existing algorithms is not strong and the performance of existing algorithms is often different due to the variation of research object problems. In ensemble learning, there is no "one-size-fits-all rule" that can be used in all scenarios. The most important goals are the combination of training data, sub-learning machines, selection algorithms, and integration algorithms. To improve the overall performance of the integrated learning machine, the research of neural network integration should mainly focus on two aspects: the accuracy and diversity of individual networks. Notably, from the perspective of the composition of integration, the choice of individual network has a great impact on both aspects.

Previous studies have proved that it may not be the best approach to integrate all sub-classifiers. On the one hand, there are sub-classifiers with both excellent and poor performance among the candidate individual sub-classifiers, with the latter leading to the performance degradation of the integrated model. On the other hand, the "repeated" integration of some sub-classifiers that produce identical or similar results will reduce the differences among sub-classifiers, which will lead to their declined ability to correct false classifications. To increase the overall accuracy of ensemble learning, the error irrelevance among sub-learning machines is more important than selecting a particular class of result integration algorithms; adjusting the parameters of the sub-learning machine may be effective for neural network integration. By using different neural network topologies (number of layers or nodes), neuron weights, objective functions, and other parameters, different sub-neural networks are trained to form an integrated neural network. Therefore, to further improve the generalization performance of the integrated model, one of the aims of this paper is to find the means to take into account the accuracy and difference between the sub-classifiers to screen those participating in the integration, that is, the selection strategy of the integration.

Regarding the efficiency of ensemble learning, one of the most obvious problems of the ensemble algorithm is the introduction of multiple sub-learning machines, which requires several times the computation demand of a single learning machine mode. Machine learning algorithms are inherently time-consuming, while the superposition of multiple learning machines will bring significant performance problems. To deal with the same machine learning problem, the computational complexity of an integrated learning algorithm does not change much compared with the algorithm of a single learning machine, but increases several times in terms of time, which is an obvious shortcoming of integrated learning. In certain real-time systems, this can become a fatal problem in practical applications. The algorithmic idea of ensemble learning can also be regarded as a dive-and-conquer strategy, which divides complex problems into several simple problems, then collects the solutions and obtains the final result through simple methods such as voting, arithmetic average and weighted average. This kind of thinking and framework coincides with many current parallel computing frameworks. Parallel computing technology is a direct and effective way to improve computing efficiency and has been applied to the learning algorithm of a single learning machine. Ensemble learning has two typical training architectures, namely, serial training architecture and parallel training architecture, while bagging and boosting are the typical representatives of these two methods. Inevitably, in ensemble learning, the framework in which each sub-learning machine is independently trained and applied is highly suitable for parallel computing. Therefore, to further improve the learning efficiency of integrated models, another principal aim of this paper is to design parallel training algorithms based on mature distributed architectures, that is, the integration of parallel training algorithms and distributed architectures.

### SEAD-PL model

#### Methodology framework of SEAD-PL

**Figure 1:**
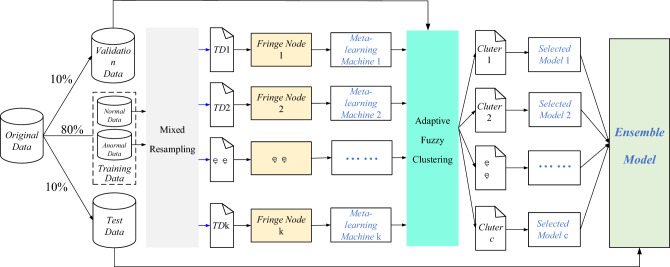
Framework of the proposed SEAD-PL approach.

The framework of the SEAD-PL method proposed in this paper is shown in Fig. 1, which includes three main modules: the processing module of the training data, the parallel training module of the base classifiers, and the selection module of the integrated model. First, the original data is divided into three parts: training set, validation set and test set. The training set is taken to train the base classifiers, which need to be processed by classification mixture resampling; the validation set is used to measure the differences between the base classifiers; and the test set is employed to evaluate the performance of the integrated model, for which no data processing is required. Secondly, the processed dataset is distributed to each distribution node for the parallel training of base classifiers. Finally, the trained base classifiers are verified by the validation set, and selective ensemble based on clustering is performed by considering the performances of both accuracy and variance, to constitute an integrated model for anomaly detection.

#### Blended resampling of unbalanced training data

**Figure 2:**
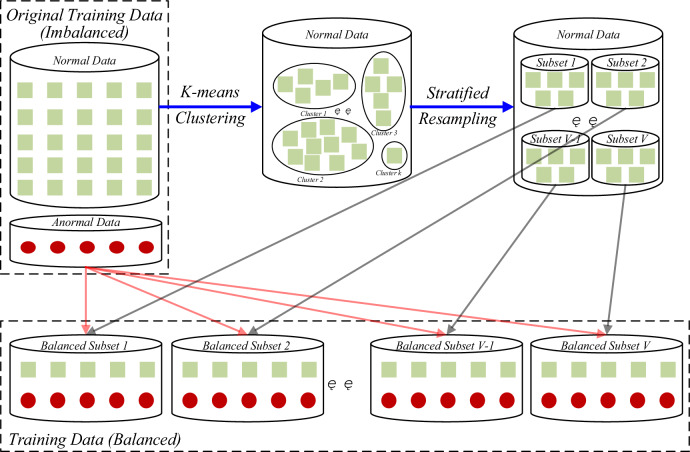
Hierarchy-based balanced subset construction.

So as to enhance the training effect of the simple base classifier and attenuate the unbalanced effect of the original training data, the unbalanced data are firstly preprocessed. The novelty of the proposed method in this subsection is that not only all original samples are retained while no new samples are introduced but also the original samples are re-processed by dividing them into a number of subsets, TD1, TD2, …, TDK, to match the subsequent parallel training step. This method consists of two main steps: (1) The multiclass is divided into a number of subclasses with the same number of samples; (2) Each subclass and lesser class is combined to form a balanced subset. An unbalanced dataset {X_N_, X_P_} is given, where X_N_ denotes multi-class and X_P_ denotes lesser class. As presented in Fig. 2, the balanced subset construction process of the hybrid sampling method based on hierarchical partitioning is divided into the following three steps:

Step 1 (Multi-class clustering): cluster the multi-class data into k classes via the K-means clustering method, denoted as {Z_1_, Z_2_, …, Z_k_}.
Step 2 (Stratified sampling): perform stratified sampling for each class cluster obtained in the first step, such that the number of samples in each subclass constituted by sampling is the same as the number of samples in the lesser class. Specifically, assume 100 samples and 10 samples from the multiclass and the lesser class, respectively: (1) Cluster the multi-class samples into three class clusters of numbers 50, 30 and 20, respectively, by step one. (2) Determine the number of balanced subsets to be constructed according to the number of parallel predetermined distribution nodes assuming 10, and then take 5(= 50/10), 3(= 30/10) and 2(= 20/10) samples, respectively, from the three clusters each time and form a subclass from a total of 10 obtained samples, which will yield a total of V subclasses.
Step 3 (Construction of balanced subsets): sequentially combine the V subclasses obtained from Step 2 with the lesser class X_P_ to form a balanced subset X_i_. A total of V balanced subsets can thus be obtained, each containing the same number of multiclass samples and lesser class samples.

#### Parallel training of base classifiers based on mapreduce

**Figure 3:**
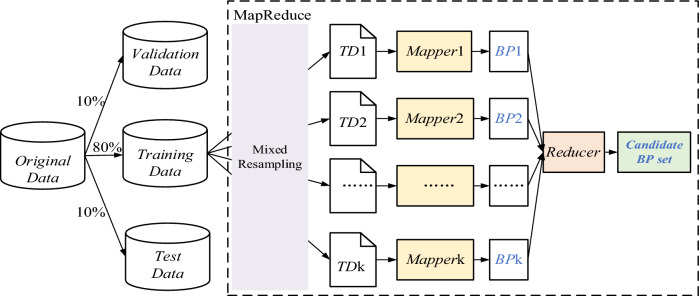
MapReduce framework for the parallel training of base classifiers.

The execution process of MapReduce consists of two key operations, Map and Reduce, both managing data in the form of key-value pairs. As shown in Fig. 3, the MapReduce architecture deployed in this paper has a pre-processed balanced sub-training set as its input and a number of trained base classifiers as its output. The base classifier chosen in this paper is the BP neural network.

First, the training set is divided into k equal-sized copies for the parallel training of k base classifiers by the hybrid sampling method described in “SEAD-PL model” section. The k sub-training sets are assigned to different mappers and the individual mappers are deployed on different computing nodes. Meanwhile, to increase the variability of individual base classifiers, the initial weights, the number of hidden layers, the number of nodes in each layer, and the node output function of the BP neural network on each mapper are randomly initialized and set at random. Each mapper trains the base classifiers in parallel according to the obtained training set. Once the parallel training of each mapper has been finished, the trained BP subclassifiers are merged into the candidate set of base classifiers by the reducer to prepare for further ensemble selection.

Here, Ts denotes the time to obtain the integrated model in an ordinary serial computing environment and to complete the detection computation on the test dataset ‘Datas’, while T_p_ denotes the time to perform the same operation in a parallel computing environment. Both T_s_ and T_p_ consist of the training time t_t_, the clustering time t_c_ and the detection time t_s_, and these two parameters can be expressed as:

1 $$T_{s} = \lambda \cdot t_{t} + t_{c} + t_{s}$$

2 $$T_{p} = \lambda \cdot \frac{{t_{t} }}{s} + t_{c} + \frac{{t_{s} }}{s} + t_{d}$$

where s represents the parallelism of the parallel computing environment, i.e., the maximum number of computing entities that can be processed in parallel for the same task, that is, the number of training iterations. In a parallel computing environment with multiple computing nodes, s means the total number of computing nodes, and t_d_ is the time loss for communication and data exchange between the nodes in the parallel environment. This value is closely related to the bandwidth, IO capability and the amount of exchanged data of the parallel computing environment. Accordingly, the time compression rate can be defined as:

3 $$S_{r} = \frac{{T_{s} }}{{T_{p} }}$$

Therefore, *S*_*r*_ < 1 and it decreases when s gradually increases, i.e., when the number of computing nodes in the parallel computing environment reaches a certain value, using the parallel architecture can improve the overall efficiency of the algorithm. However, not all steps can be parallelized and there is the additional issue of data transmission consumption; it is impossible for the parallel computing environment to make the acceleration equivalent to the number of computing nodes; *S*_*r*_ will increase continuously with *s*, but this growth will be slower than that of s. Besides, the growth of *S*_*r*_ is closely related to the divisibility and parallelizability of the problem.

**Figure 4:**
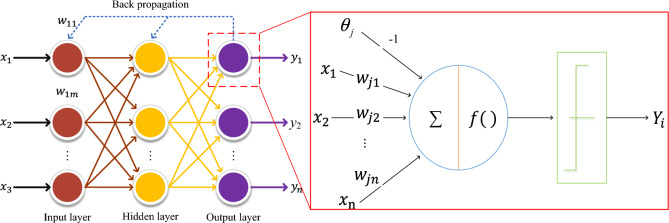
Schematic diagram of the three-layer BP neural network.

For the base classifier, the BP neural network is chosen in this paper because of its excellent ability to deal with nonlinear features. At the same time, to avoid the meta-learning being overly complex, the BP neural network is designed with a single hidden layer, i.e., a three-layer BP neural network is used as the base classifier, as shown in Fig. 4. Each BP network is trained using a process common with the gradient descent method.

#### Selective ensemble based on adaptive clustering

After parallel training, once the candidate base classifiers have been obtained, we have to try to select the individuals with good results and large differences among them to participate in the ensemble model. In this paper, we propose to cluster the base classifiers with small differences into a single cluster, and the base classifiers in different clusters can be considered to have large differences among themselves. Thus, we can obtain better integrated detection results by selecting the best-performing base classifiers in the clusters to participate in the ensemble.

In order to realize the clustering of individuals in the candidate set, the first step is to define the "distance" between individuals that represents the differences between them. We use the pairwise variability criterion to measure this difference. In this criterion, assuming two base classifiers, C_i_ and C_j_, for the same validation set V where the total sample size of the validation set is v, the variability in performance between the two is reflected in the overall classification accuracy of the two out of all samples v in the validation set V. Here, v_ij_ and v_00_ denote the number of samples that are both correctly classified and both incorrectly classified by classifiers C_i_ and C_j_, respectively, on the validation set V; v_i0_ denotes the number of samples that are correctly classified by classifier C_i_ and incorrectly classified by C_j_, and v_0j_ denotes the number of samples that are correctly classified by classifier C_j_ and incorrectly classified by C_i_. This leads to the following relation:

4 $$v_{ij} + v_{i0} + v_{0j} + v_{00} = v$$

Furthermore, the inconsistency measure is employed to directly characterize the differences between the two classifiers:

5 $$INC_{ij} = \frac{{v_{i0} { + }v_{0j} }}{{v_{ij} + v_{i0} + v_{0j} + v_{00} }} = \frac{{v_{i0} { + }v_{0j} }}{v}$$

If all candidate k base classifiers are considered, the average of the inconsistency measures can be further regarded:

6 $$\overline{INC} = \frac{2}{k(k - 1)}\sum\limits_{i = 1}^{k - 1} {\sum\limits_{j = i + 1}^{k} {INC_{ij} } }$$

INC_ij_ can be taken as the distance between base classifiers C_i_ and C_j_, which can be understood as the average of distances between all base classifiers $\overline{INC}$. Moreover, the variability of different two pairs of base classifiers can be compared:

7 $$\Delta INC{ = }\left| {INC_{ij} - INC_{mn} } \right| = \left| {\frac{{v_{i0} - v_{m0} }}{v}} \right| + \left| {\frac{{v_{0j} - v_{0n} }}{v}} \right| = \frac{1}{v}(\left| {v_{i0} - v_{m0} } \right| + \left| {v_{0j} - v_{0n} } \right|)$$

After defining the "distance" between the base classifiers, they can be further clustered and the most important task will be the determination of the number of clusters. To avoid the transitional dependence on human experience, this paper adopts the adaptive fuzzy approach to determine the optimal number of clusters.

Adaptive fuzzy clustering methods generally identify the optimal number of clusters, c_best_, based on the exhaustive search strategy of fuzzy clustering effectiveness index between [c_min_, c_max_], where obviously c_min_ < c_best_ < c_max_.

After determining the minimum and maximum number of clusters, c_best_ needs to be established by an exhaustive search strategy based on fuzzy clustering validity metrics between [c_min_, c_max_]. The smaller the fuzziness, the more reliable the clustering results will be. Meanwhile, a candidate set that can be better categorized should be tight within each cluster and as sparse as possible between clusters. In this paper, we implement the S.H. Kwon validity index^43^:

8 $$V_{Kwon} (U,O,c) = \frac{{\sum\nolimits_{i = 2}^{{c_{\max } }} {\sum\nolimits_{j = 1}^{L} {u_{ij}^{m} \left\| {o_{i} - l_{j} } \right\|^{2} } } + \frac{1}{c}\left\| {o_{i} - \overline{o}} \right\|^{2} }}{{\mathop {\min }\limits_{i \ne j} \left\| {o_{i} - o_{j} } \right\|^{2} }}$$

where *U* denotes the affiliation matrix, *O* denotes the cluster center matrix, *c* denotes the number of clusters, n denotes the total number of candidate subsets, *u*_*ij*_ is an element in the affiliation matrix* U*, m denotes the fuzzy factor, *o*_*i*_ is an element in the cluster center matrix *O*, *l*_*j*_ is an element in the candidate set, and $\overline{o}$ denotes the average value of all cluster centers.

The combined relationship between intra-cluster tightness and inter-cluster sparseness is represented by the numerator and denominator ratios. Obviously, in the clustering process, it is assumed that the tighter the intra-cluster and the sparser the inter-cluster, the better the final clustering effect. Therefore, the smaller the value of this indicator, the better the clustering effect of the dataset. Importantly, the number of clusters is optimal when the value of the indicator is the smallest. After determining the number of clusters, c_best_ can be clustered for all base classifiers, and from each cluster, the base classifier that performs best in the validation set is selected to participate in the integrated model. The final result of the integrated model is obtained by voting.

## Experimental methodology

### Experimental design

#### Benchmarked datasets

In this paper, we utilize six datasets for experimental study, as shown in Table 1. Two datasets, Page Blocks and Satimage-2, are derived from the UCI database, which are anomaly detection datasets containing true anomaly semantics; KDDCUP 99 is a classic open-source dataset for cybersecurity anomaly detection, with some of its data randomly selected due to its overall volume of 4.9 million entries and limited experimental computational power; BATADAL is an industrial control system dataset; the Water Storage dataset comprises network traffic data captured by a Mississippi State University lab on water storage tanks through a Supervisory Control and Data Acquisition (SCADA) system; Power is an electric power system attack dataset, which is jointly collected by Mississippi State University and Oak Ridge National Laboratory. Overall, these six datasets, with data volumes ranging from 2,579 to 449,919 entries and anomaly rates from 1.2 to 35.3%, are appropriate to effectively evaluate the robustness of the proposed method.

**Table 1:** Details of the real-word experimental dataset.

Dataset	Instances	Features	Abnormal rate (%)
Page blocks	4982	10	2.0
Satimage-2	5803	36	1.2
KDDCUP99	60,839	38	14.6
BATADAL	13,938	43	1.6
SWaT	449,919	51	12.1
Power	5276	129	35.3

We further used six other benchmarked datasets extracted from the UCI Machine Learning Repository. Table 2 summarizes these six datasets, with IR (Imbalance Rate) ranging from 1.38 to 194.46.

**Table 2:** Characteristics of the UCI Machine Learning Repository datasets.

Dataset	Instances	Features	Abnormal rate (%)
Liver	345	6	1.38
Vehicle	846	17	2.88
Hepatitis	80	19	5.15
Balance	625	4	11.76
Derma	358	34	16.90
Yeast	1484	8	48.47

#### Evaluation metrics

First, we measure the accuracy of anomaly detection algorithms using the AUC, i.e., the area under the ROC (Receiver Operating Characteristic) curve, which is a dominant metric in the field of anomaly detection research and is well suited for algorithm performance evaluation on datasets with different anomaly ratios. The optimal anomaly detection algorithm has an AUC value of 1, with larger AUC values indicating higher accuracy of the algorithm. Second, we further evaluate the performance of the algorithms using the Friedman test, a commonly used metric to enable the performance comparison of multiple algorithms on multiple datasets. In terms of the temporal and spatial cost of the algorithms, we mainly compare the time required for training the algorithms and the memory consumption. We average the results of each set of experiments five times.

#### Experimental design

Two aspects of the experiment are designed to achieve a more comprehensive comparative analysis.

Firstly, the integrated detection method proposed in this paper is evaluated against the commonly used anomaly detection methods, and the AUC values of the anomaly detection results of different algorithms are compared. The most commonly compared anomaly detection methods are further subdivided into two types:

- robustness-enhanced non-integrated anomaly detection methods, such as those based on the robust OCSVM (ROCSVM)^44^ and the density-weighted SVDD (DWSVDD)^45^. These methods represent two trends in robustness enhancement techniques: ROCSVM replaces the loss function of the original model using a ramp loss function to reduce the negative impact caused by anomalous samples, while DWSVDD improves algorithmic robustness by assigning a different weight to each sample, where the anomalous samples are given a smaller weight than the normal samples. Essentially, these methods improve anomaly detection through cost-sensitive performance without using ensemble.
- existing anomaly ensemble methods, including RB (Rotated bagging)^46^ and CARE (The cumulative agreement rate ensemble)^27^. These two methods are currently the more popular anomaly ensemble algorithms. The former combines the advantages of parallel ensemble and sequence ensemble by designing a two-stage combination technique within the learning framework of the FG method, while the latter adds a stochastic projection trick before feature selection to further enhance the ensemble diversity.

Secondly, to validate the innovative contribution of this paper, three sets of truncated experiments are designed: comparative validation for data preprocessing, parallel training architecture, and selective ensemble. In the data preprocessing stage, the experimental results after no preprocessing, random sampling and the hierarchical sampling proposed in this paper are separately evaluated. In the training modes, the temporal and spatial costs are compared between two modes, namely, parallel and serial. In the ensemble strategies, the differences in the experimental results of a single un-integrated (the best-performing base classifiers), fully-integrated and selective ensemble are compared.

Finally, *x* the number of parallel nodes is compared, which is the main factor affecting the methodology of this paper. This is performed by increasing and decreasing the number of parallel nodes by 50% relevance to the original parameters.

#### Experimental parameters

The experiment is based on the Hadoop environment, with 50 nodes set up (each group of experiments may not be all called), single node configuration 8-core CPU, 16 Gb RAM, while ignoring the impact of node stability and node performance differences. To ensure that each node has sufficient data for base classifier training, the capacity is different for each dataset, and at the same time, the richness of the candidate set integrated after parallel training is considered. Each group of experiments is conducted according to the data sample capacity to call no less than 3 nodes for parallel training, but for the same dataset of multiple experiments, the number of nodes are kept unchanged.

The parameter setting of SEAD-PL needs to be considered from three aspects: the parameters of base classifier BP (number of hidden layers, number of hidden nodes, initial weight, learning rate), the number of parallel training nodes, and the number of selected integrated clusters.

For the parameters of base classifier BP, this paper mainly deals with text data. The deep network efficiency is low and the cost performance is not high, so the single hidden layer setting is adopted. In the hidden layer nodes, the number of nodes is too small, the effective information obtained by the neural network is insufficient, the accuracy is correspondingly low, and the convergence speed of the network is too slow. An excessive number of hidden layer nodes, complex network topology and long learning time will easily lead to overfitting. To increase the difference between the base classifiers, a random value between 0 and the number of nodes in the input layer is set. Among them, the number of nodes in the input layer is consistent with the feature dimension extracted from the dataset.

To some extent, the initial weight determines the convergence range and efficiency of the training results. There are three common ways to set the initial weight: taking random values in a certain interval; setting random values in small intervals near 0; initialize the input layer to the hidden layer with small random numbers, and initialize the hidden layer to the output layer with − 1 or 1. Considering increasing the randomness of the base classifier and its training efficiency, the third method is adopted in this paper.

The learning rate has a great influence on the BP training time: if its value is too small, the convergence is slow, while if it is too large, it will cause shock or even non-convergence. The adaptive learning rate is helpful in shortening the training time; it can be adjusted globally or differentially. The former is more commonly used, which can be implemented by a variably long learning rate algorithm, an increasing and decreasing adjustment algorithm, and a hierarchical algorithm. This paper adopts a segmented learning rate adjustment method, such as:

9 $$\eta^{\prime} = \left\{ \begin{array}{ll} 0.7\eta & \quad CE^{\prime} > CE * 1.04 \hfill \\ 1.05\eta & \quad CE^{\prime} < CE \hfill \\ \eta & \quad {\text{ others }} \hfill \\ \end{array} \right.$$

*CE* represents the expected output cross entropy, and $CE^{\prime}$ represents the actual output cross entropy.

In terms of the number of parallel training nodes, due to the hierarchical sampling strategy adopted in this paper, the number of copies divided, the number of parallel nodes and the number of BP base classifiers in the overall dataset are consistent. Based on this setting, we should consider the number of copies that the overall dataset is divided into. The purpose of stratified sampling is to balance datasets, so the number of divided copies of the overall dataset *V* = *N*_*max*_/*N*_*min*_, where *N*_*max*_ and *N*_*min*_ represent the number of majority and minority samples, respectively. Obviously, when these two are not evenly divided, *V* can be taken forward.

In terms of selecting the number of integrated clusters, since the adaptive clustering method is adopted in this paper, it is necessary to set the minimum cluster number c_min_ and the maximum cluster number c_max_, then solve the optimal cluster number c_best_ according to Formula (8). The minimum cluster number c_min_ is set to two. Apparently, less than two clusters are not necessary for clustering. To improve the computing efficiency, the maximum cluster number c_max_ can be set to n, where n represents the number of candidate subsets.

A more comprehensive comparative analysis involving the comparison algorithms can be performed by setting up multiple parameter combinations from each comparison algorithm. Specifically, the ROCSVM anomaly ratio α1, and the number of nearest neighbors k in DWSVDD, CARE and RB are selected from the sets {0.01, 0.03, 0.05, 0.07, 0.09} and {2, 4, 6, 8, 10}, respectively.

### Results and discussion

#### Performance comparison of the overall detection effect 

##### Comparison with certain robust non-integrated anomaly detection methods

In order to evaluate the anomaly detection effectiveness of the SEAD-PL method proposed in this paper, we compare it with two existing robust non-integrated anomaly detection methods. These algorithms are popular and effective for reducing the interference of anomalous samples. Table 3 lists the AUC results of the comparison algorithms in the form of multiple parameter combinations, as well as the AUC results of our SEAD-PL algorithm.

**Table 3:** Comparison of experimental results between SEAD-PL and two robust non-integrated anomaly detection algorithms.

Method	Page Blocks	Satimage-2	KDDCUP99	BATADAL	SWaT	Power	Mean
ROCSVM (α1 = 0.01)	0.8537	0.5324	0.6676	0.8422	0.9242	0.9177	0.7896
ROCSVM (α1 = 0.03)	0.8645	0.5190	0.6324	0.8636	0.9166	0.9262	0.7871
ROCSVM (α1 = 0.05)	0.8635	0.5263	0.6887	0.8362	0.9263	0.9136	0.7924
ROCSVM (α1 = 0.07)	0.9066	0.5148	0.6875	0.8763	0.9376	0.9261	0.8082
ROCSVM (α1 = 0.09)	**0.9108**	0.5293	0.6772	0.8881	0.9288	0.9182	0.8087
DWSVDD (k = 2)	0.7302	0.6081	0.5364	0.7472	0.9247	0.9074	0.7423
DWSVDD (k = 4)	0.7896	0.6015	0.5636	0.7569	0.9296	0.9033	0.7574
DWSVDD (k = 6)	0.7919	0.6136	0.6013	0.7820	0.9208	0.9050	0.7691
DWSVDD (k = 8)	0.7653	0.6131	0.6132	0.7713	0.9311	0.9128	0.7678
DWSVDD (k = 10)	0.7221	0.6525	0.5987	0.7755	0.9255	0.9127	0.7645
SEAD-PL	0.9091	**0.7782**	**0.8016**	**0.8933**	**0.9886**	**0.9506**	**0.8869**

Significant values are in bold.

As can be seen from Table 3, SEAD-PL exhibits the best performance on all but one experimental dataset (Page Blocks) when compared to the two robust non-integrated anomaly detection algorithm methods. On the Page Blocks dataset, SEAD-PL ranks second behind ROCSVM (α1 = 0.09) but exhibits very similar results. For example, the AUC value of SEAD-PL on the Page Blocks dataset is 0.9091, while the optimal AUC value is 0.9208. This suggests that the SEAD-PL method proposed in this paper is either significantly better than the comparison algorithms or close to being the optimal algorithm. More notably, anomaly detection is significantly improved on 5/6 datasets. Finally, by observing Table 3, it can be deduced that SEAD-PL exhibits the best average performance and can improve the average AUC value by more than 8%.

It should be noted that the performance of SEAD-PL is second to ROCSVM (α1 = 0.09) on the Page Blocks dataset, which has only 10 eigenvectors (the least among all datasets), making it a typical low-dimensional data. In SEAD-PL, the Base Classifier is BP, which is more suitable for high-dimensional data detection and has outstanding nonlinear mapping ability. ROCSVM replaces the loss function of the original model using a ramp loss function to reduce the negative impact caused by anomalous samples, improving anomaly detection performance through cost-sensitivity. When the dimensionality is low, the cost-sensitive computation can achieve a more precise result than neural networks with an appropriate parameter setting, since its computational complexity is low.

**Figure 5:**
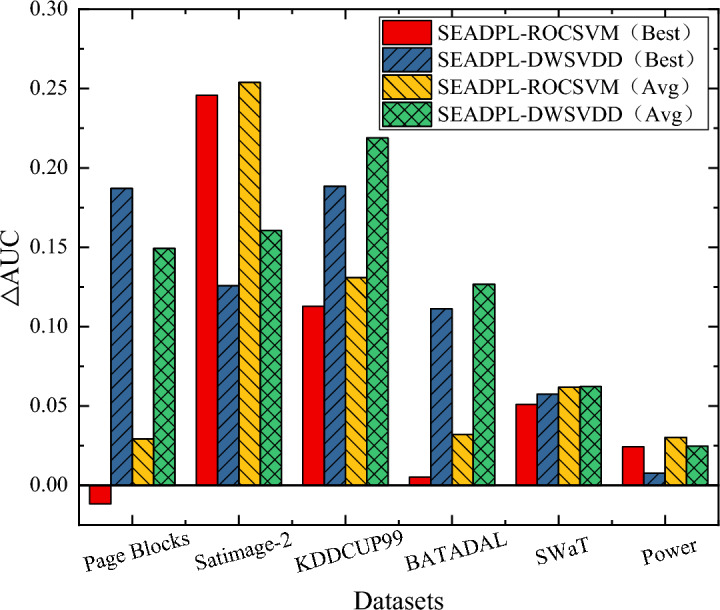
AUC difference analysis between SEAD-PL and two robust non-integrated anomaly detection algorithms.

In Fig. 5, we compare and analyze the AUC results of the proposed SEAD-PL with the AUC results of the two comparison algorithms, where the AUC values of these algorithms are selected from the best AUC results in Table 2. As can be seen from the figure, SEAD-PL demonstrates better performance than the comparison algorithms on 5/6 experimental datasets. Moreover, the average AUC improvement of SEAD-PL ranges between 2.47 and 25.38%.

The phenomenon observed above is explained by the following analysis: the comparison algorithms use the technique of sample weighting to improve the robustness of the algorithms; however, their sample weighting process is independent of the subsequent anomaly scoring, and in the absence of data labels, this independent training mode is unable to obtain the optimal representation of samples. The SEAD-PL method proposed in this paper is based on integrated learning to reduce the influence of anomalous samples on model training and adopts the idea of stratified sampling.

In addition, SEAD-PL performs better on experimental datasets with high anomaly ratios and feature dimensions (e.g., the SWaT and Power datasets), while the performance enhancement is more obvious on small-scale low anomaly ratio datasets (e.g., the Satimage-2 dataset). In summary, the method proposed in this chapter shows a more robust performance improvement compared to the popular robust single-classification anomaly detection algorithms.

##### Comparison with the current ensemble anomaly detection methods

In order to enable comparative analysis with similar algorithms, this subsection takes the SEAD-PL algorithm and existing anomaly ensemble algorithms for comparison. Unlike the previous experimental setup, all compared algorithms in this subsection, including the SEAD-PL algorithm, are run independently 10 times due to the presence of a randomness module. The AUC results shown in Table 4 are all averages of the results from 25 experiments.

**Table 4:** Comparison of experimental results between SEAD-PL and the latest integrated anomaly detection algorithm.

Method	Page Blocks	Satimage-2	KDDCUP99	BATADAL	SWaT	Power	Mean
CARE (k = 2)	0.7136	0.7557	0.7676	0.7521	0.9124	0.9077	0.8015
CARE (k = 4)	0.7735	0.7533	0.7236	0.7636	0.9262	0.8926	0.8055
CARE (k = 6)	0.7904	0.7611	0.7135	0.7362	0.9265	0.9063	0.8056
CARE (k = 8)	0.8070	**0.7869**	0.7857	0.7763	0.9267	0.9161	0.8331
CARE (k = 10)	0.8076	0.7459	0.7277	0.7818	0.9318	0.9228	0.8196
RB (k = 2)	0.7307	0.7036	0.6463	0.8427	0.9347	0.8947	0.7921
RB (k = 4)	0.7661	0.7150	0.6766	0.8596	0.9369	0.9203	0.8124
RB (k = 6)	0.8183	0.7372	0.7031	0.8208	0.9308	0.9105	0.8201
RB (k = 8)	0.8459	0.7126	0.7312	0.8713	0.9371	0.9117	0.8350
RB (k = 10)	0.8520	0.6935	0.6977	0.8557	0.9355	0.9202	0.8258
SEAD-PL	**0.9091**	0.7782	**0.8016**	**0.8933**	**0.9886**	**0.9506**	**0.8869**

Significant values are in bold.

From Table 4, SEAD-PL shows the best detection results on 5/6 experimental datasets. Specifically, on the Satimage-2 dataset, the AUC difference between SEAD-PL and the optimal comparison algorithm, CARE (k = 8), is only 0.0087. Meanwhile, on this dataset, SEAD-PL significantly outperforms the comparison algorithms for all other parameters of CARE and all parameters of RB. It is clear from the last column that SEAD-PL can increase the average AUC value of detection over the existing integrated algorithms by more than 5%.

It should be noted that the performance of SEAD-PL is after the CARE (k = 8) in the Satimage-2 dataset. The abnormal rate of Satimage-2 is only 1.2, the least among all datasets, making it a typical high-imbalanced data. In SEAD-PL, the blended resampling strategy is used to rebalance the dataset. However, when the dataset is highly imbalanced, very few abnormal instances are assigned to each compute node. That is to say, insufficient sample reduces the learning efficiency of base classifiers, and although integration operation is followed, it still cannot get the best effect. CARE adds a stochastic projection trick before feature selection to further enhance the diversity of the ensemble. Consequently, it is more effective to deal with high-imbalanced data when the parameter setting is appropriate (k = 8).

**Figure 6:**
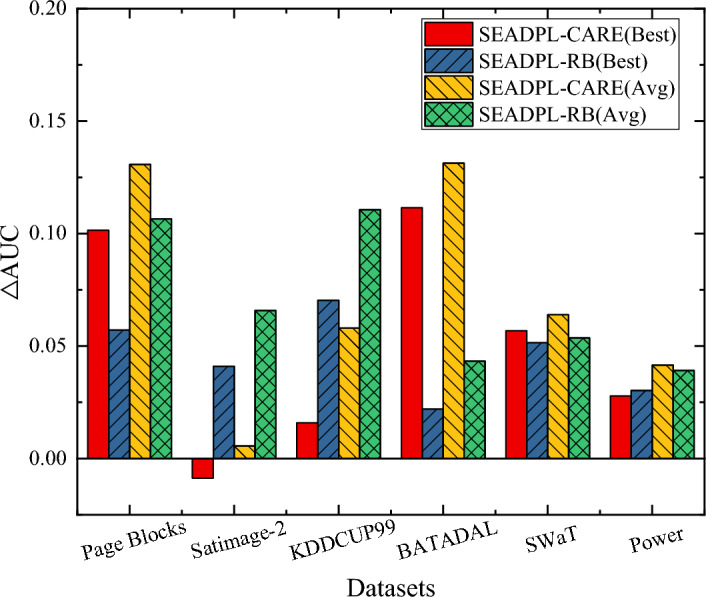
AUC difference analysis between SEAD-PL and the latest integrated anomaly detection algorithms.

Figure 6 depicts the average AUC difference between SEAD-PL and each comparison algorithm. For convenience, we refer to the average AUC values collectively as AUC values in subsequent sections. The SEAD-PL method proposed in this paper shows the best performance on the SWaT dataset, with an AUC value of 0.9886. This suggests that the use of an ensemble-based learning framework helps improve the performance of common anomaly detection algorithms, and that the selective ensemble technique proposed in this paper is useful for improving the existing anomaly ensemble algorithms. In addition, the △AUC values of SEAD-PL are all positive. Overall, SEAD-PL has more stable performance compared to popular anomaly ensemble algorithms.

As can be seen from Fig. 6, SEAD-PL achieves an AUC performance improvement on most of the experimental datasets compared to all other algorithms. Specifically, SEAD-PL outperforms the RB algorithm on 6/6 experimental datasets and the CARE algorithm on 5/6 experimental datasets. From an overall perspective, when compared to the CARE algorithm, CARE is less stable, although it slightly outperforms SEAD-PL on the Satimage-2 dataset. In addition, as shown in Fig. 6, the average AUC appreciation of SEAD-PL on the 10 experimental datasets ranges from 1.76 to 13.07%. To sum up, compared with existing ensemble-based anomaly detection algorithms, the training method proposed in this paper that considers robustness enhancement can realize the further enhancement of existing anomaly ensemble algorithms.

##### Running time and memory requirements

Next, to test the cost of different algorithms on different datasets, the running time and memory are compared. These factors are averaged with different parameters for ROCSVM, DWSVDD, CARE and RB. The comparison results for computational efficiency are shown in Table 5.

**Table 5:** Running time consumption of the compared algorithms (seconds).

Dataset	ROCSVM	DWSVDD	CARE	RB	SEAD-PL
Page blocks	2.98 (3)	3.79 (5)	3.56 (4)	2.67 (2)	**1.76 (1)**
Satimage-2	5.25 (5)	4.88 (3)	4.92 (3)	4.06 (2)	**2.44 (1)**
KDDCUP99	20.37 (5)	17.78 (4)	14.66 (3)	15.37 (3)	**11.73 (1)**
BATADAL	6.23 (5)	6.09 (4)	5.56 (2)	5.58 (3)	**5.12 (1)**
SWaT	45.94 (5)	35.96 (3)	38.79 (4)	33.13 (2)	**28.59 (1)**
Power	5.46 (5)	4.38 (3)	5.09 (4)	4.21 (3)	**2.18 (1)**
Average ranking	4.67 (5)	3.67 (4)	3.33 (3)	2.5 (2)	**1.0 (1)**

Significant values are in bold.

The experimental findings show that the proposed SEAD-PL algorithm achieves the highest average ranking of time consumption and the best comprehensive computational efficiency on six datasets. The RB algorithm has slightly inferior comprehensive computational efficiency on three datasets compared to EGSCC, and the difference between them is not obvious on the BATADAL dataset. The ROCSVM algorithm consistently has the largest computational consumption. The gap between CARE and DWSVDD is not large. The average potential ranking of CARE is 3.33, which is the third among the seven algorithms, while DWSVDD has an average ranking of 3.67, only surpassing that of ROCSVM. The SEAD-PL algorithm uses parallel training strategy to avoid unnecessary computations. Table 6 shows the memory consumption of each algorithm.

**Table 6:** Memory consumption of the compared algorithms (MB).

Dataset	ROCSVM	DWSVDD	CARE	RB	SEAD-PL
Page blocks	1.66 (4)	**1.28 (1)**	1.97 (5)	1.54 (2)	1.59 (3)
Satimage-2	1.96 (4)	**1.44 (1)**	2.12 (5)	1.75 (3)	1.66 (2)
KDDCUP99	14.52 (4)	13.89 (2)	16.85 (5)	**13.66 (1)**	14.07 (3)
BATADAL	25.35 (4)	**23.74 (1)**	30.77 (5)	24.88 (2)	25.12 (3)
SWaT	60.83 (3)	55.65 (2)	30.77 (5)	**51.03 (1)**	62.24 (4)
Power	1.81 (4)	**1.30 (1)**	2.08 (5)	1.69 (3)	1.62 (2)
Average ranking	3.83 (4)	**1.33 (1)**	5 (5)	2 (2)	2.83 (3)

Significant values are in bold.

As can be seen from the data, DWSVDD consumes the least memory, followed by RB, while CARE is the costliest. The experimental results indicate that the proposed SEAD-PL algorithm has an average level of memory consumption on 6 datasets. Furthermore, the proposed SEAD-PL algorithm has slightly inferior comprehensive memory consumption on four datasets than RB, and it exceeds that of RB algorithm on the Satimage-2 and Power datasets. There is an obvious difference between SEAD-PL and ROCSVM: the average potential ranking of ROCSVM is 3.83, which is the fourth among the five algorithms, while SEAD-PL has an average ranking of 2.83, which places it next to RB.

##### Generalization capability analysis

For the generalization ability measure, we use the Nemenyi test to implement a performance comparison between the SEAD-PL algorithm and four other algorithms.

As for the experimental datasets, another six datasets shown in Table 2 are added to conduct a more comprehensive analysis in addition to the datasets shown in Table 1.

First, we rank all the algorithms on each dataset: the best performing algorithm is ranked 1st and the worst performing algorithm is ranked 5th. Table 7 describes the average ranking values obtained by each algorithm on the 12 datasets, while the test results are presented to compare the different ranking results obtained by the algorithms using different parameter settings. The detailed parameter settings for each test are as follows: in test 1, k = 2, α1 = 0.09; in test 2, k = 4, α1 = 0.03; in test 3, k = 6, α1 = 0.05; in test 4, k = 8, α1 = 0.07; in test 5, k = 10, α1 = 0.09; in test 6, each comparison algorithm selects the best AUC result for the comparison; in test 7, each comparison algorithm selects the average AUC result for comparison. As can be seen from the data, SEAD-PL ranks first in each test, which indicates that this algorithm has the best overall performance compared to all other algorithms.

**Table 7:** Mean ranking results of Nemenyi test for SEAD-PL and the compared algorithms.

Test	ROCSVM	DWSVDD	CARE	RB	SEAD-PL
Test 1	3.55	4.55	3.25	3.03	1.00
Test 2	3.44	4.12	3.56	3.08	1.00
Test 3	3.23	4.40	3.69	2.97	1.00
Test 4	3.11	4.69	3.63	3.49	1.25
Test 5	3.79	4.88	2.72	3.56	1.13
Test 6	2.66	4.39	2.98	3.56	1.28
Test 7	3.31	4.30	3.64	3.13	1.00

**Figure 7:**
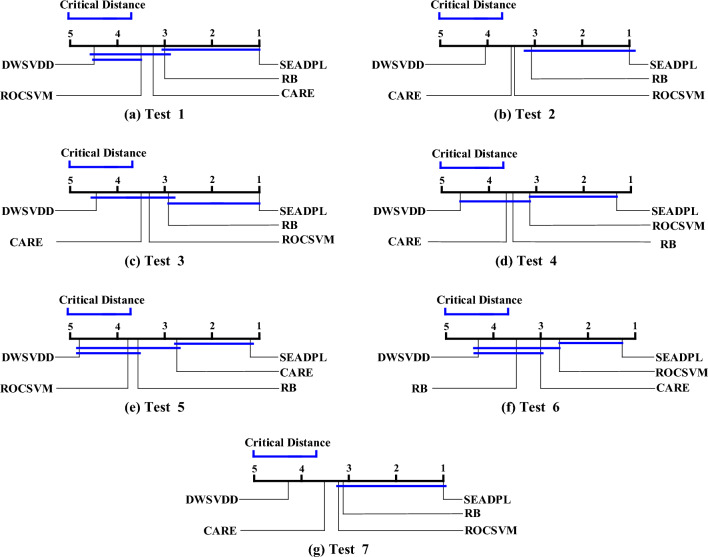
Comparison of post-hoc test results between SEAD-PL and other selected algorithms.

Second, to verify the correctness of the null hypothesis (that is, all compared algorithms perform equivalently), we first compute the FF statistic, which is obtained using the F distribution with degrees of freedom (5, 5 × 7). Specifically, let the α-quantile be 0.1, then the critical value (cα) of F (5,30) is 2.05. In this paper, cα is significantly smaller than the sorted mean value of 3, hence the null hypothesis does not hold. In other words, there is some variability in the performance of the experimentally compared algorithms in this section. Finally, to accurately assess the variability between the two algorithms, we next use the post-hoc test, in which the critical difference (CD) is calculated as follows:

10 $$CD = C_{\alpha } \sqrt {\frac{{N_{\alpha } (N_{\alpha } + 1)}}{{6N_{d} }}}$$

where Nα represents the number of algorithms and Nd represents the number of experimental datasets. In this subsection of experiments, the CD value is 1.29. If the difference in the sorting average between two algorithms is greater than or equal to CD, it means that there is a relatively significant difference between this algorithm pair. Figure 7 lists all the comparison results from the seven tests.

In each test, the SEAD-PL method achieves a significant performance improvement compared to all other algorithms. Considering the different parameter settings for each test, we can observe that the variation in the parameter values makes the anomaly detection performance of the comparison algorithms also fluctuate. Similarly, at the overall level, the integrated algorithms CARE and RB obtain results closer to the SEAD-PL algorithm, while the non-integrated algorithms both perform the worst in the tests. Therefore, we can conclude that SEAD-PL has several significant advantages over the existing anomaly detection algorithms.

#### Results of truncation experiments

##### Comparison of different data processing methods

**Figure 8:**
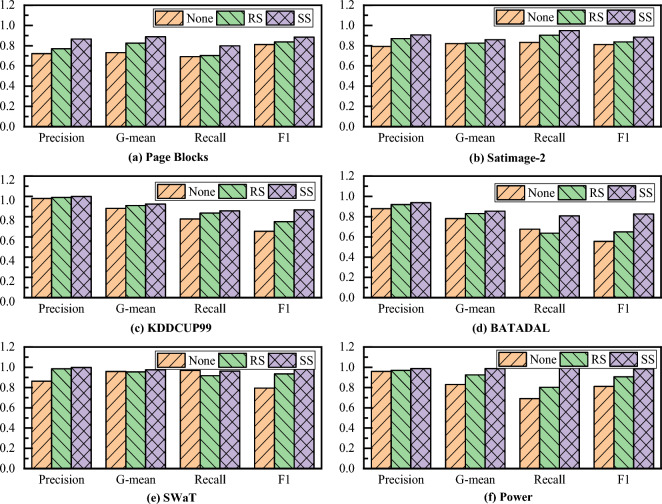
Comparison of integrated detection performance for different data processing methods.

This subsection compares the detection results using raw data (no sampling, None), Random Sampling processing (Random Sampling, RS), and the Stratified Sampling method (Stratified Sampling, SS) proposed in this paper. For the purpose of truncated validation, the strategies of subsequent parallel training and selective ensemble are the same for each experiment of data processing. Thus, the detection performance is comparatively analyzed in terms of accuracy, recall, G-mean, and F1, and the results are presented in Fig. 8.

As can be seen from Fig. 8, the integrated algorithm after stratified sampling treatment yields the best detection effect on all six datasets, which is significantly higher than that by the non-sampling and random sampling treatments. For example, the hierarchical sampling post-ensemble detection algorithm, which has a recall of about 0.94 on the KDDCUP99 dataset and about 0.98 on the other SwaT and Power datasets, can achieve roughly 20–50% increase compared to the other non-sampling and random sampling methods. This significant improvement of recall rate means that the false alarm rate of abnormal data is reduced. Moreover, the detection method can more accurately identify abnormal samples and improve the safety of the system while taking into account the real-time nature of the system and also avoiding the ineffective response of the system due to the high false alarm rate. The experimental results demonstrate the robustness of the selective ensemble method with hierarchical sampling processing, a certain migration ability between different detection tasks, and its better detection performance.

Furthermore, the G-mean of the selective integrated detection method after stratified sampling is higher than that of other treatments, e.g., its G-mean is 0.9423 on the KDDCUP99 dataset, 0.9976 on the SwaT dataset, and 0.9954 on the Power dataset, which is roughly 5–15% higher compared to other treatments. This improved G-mean value implies a significant precision enhancement while considering recall. Besides, the F1 score of the selective integrated detection method after stratified sampling is also higher than that of other data processing methods, that is, 0.8864 on the KDDCUP99 dataset, 0.9885 on the SwaT dataset, and 0.9835 on the Power dataset, which constitutes a 20–35% improvement. The F1 score as the comprehensive evaluation index of the model considers both precision rate and recall rate, such that the two reach a balance point. Therefore, this score reflects the comprehensive performance and stability of the model to a certain extent, and the larger the F1 value, the better the comprehensive performance and stability of the model, which further illustrates the advantages of our stratified sampling method.

##### Comparison of time efficiency of different training modes

**Figure 9:**
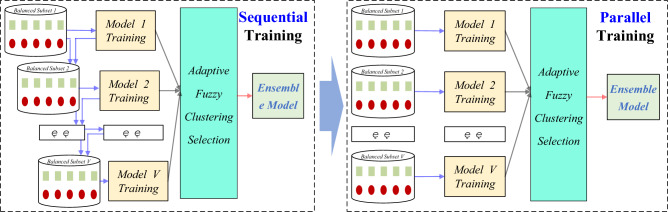
Parallel training mode vs. serial training mode.

In this paper, to improve the training efficiency of large-scale data and to overcome the performance of integrated detection algorithms in the training phase, we propose a parallel training mode based on the Mapreduce framework. To verify the advantage of the parallel training mode in terms of efficiency, in this subsection, we compare the time requirement between the parallel training mode and the serial serialized training mode (Fig. 9), then calculate the time compression rate of parallel training on each dataset based on the serialized training time using the same pre-experimentation data hierarchical sampling and subsequent selective ensemble strategies.

**Figure 10:**
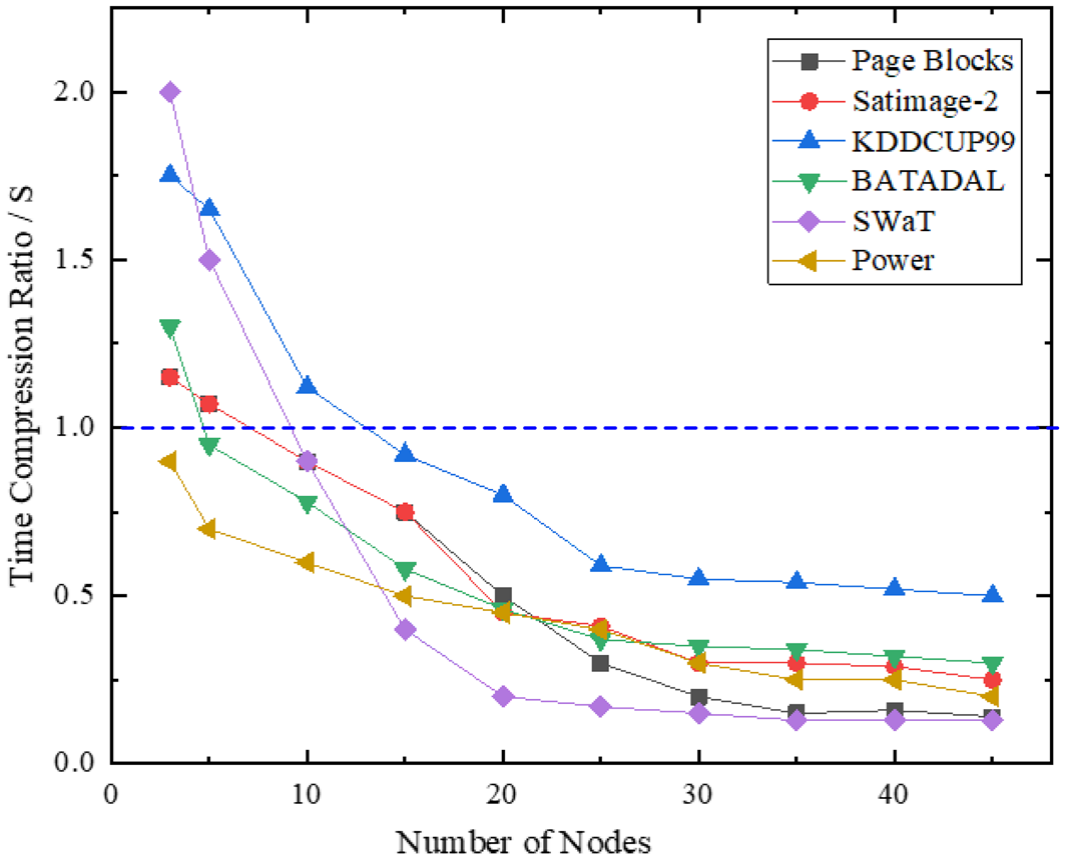
Time compression rates for parallel training sessions.

MapReduce can decompose complex or data-intensive problems into simple sub-problems, which are then parallelly processed in multiple computing nodes. The time compression rate results of the proposed method on each dataset are illustrated in Fig. 10. As can be seen from the figure, in the base classifier training session, the time compression rate using the parallel operation mode uniformly decreases as the number of parallel nodes increases. That is to say, within a certain range, the more parallel nodes are present, the more obvious the time advantage of parallel training, which is essentially due to the excellent parallel computing capability of MapReduce. Meanwhile, once the degree of parallelism has reached a certain level, by further increasing the number of nodes, the time compression rate does not change much.

For example, for the SwaT dataset, the training time decreases significantly before the number of parallel nodes reaches 20, and then the training time no longer changes much when this number continues to increase. Instead, as the parallelism increases, the amount of data transmitted to each node decreases and the transmission time shortens. This is because with an increasing number of parallel nodes, the amount of training data obtained by each node decreases, hence the base classifier requires more iterations to complete the training. This also means that the best parallel operation state has been reached at this point and its corresponding number of parallel nodes is the optimal value.

Meanwhile, for most datasets, the time compression ratio is greater than 1 when the number of parallel nodes is small, which means that the training time increases instead of decreasing. For example, for the KDDCUP99 dataset, the time advantage is not reflected in the parallel training until the number of parallel nodes reaches 10, and the largest time increase occurs when the number of nodes is 3. This is because when the degree of parallelism is not high, the time compression of parallel operation is less than that data transmission and node communication, leading to a subsequent increase in the total training time.

In summary, when the appropriate number of parallel nodes is set, parallel training can significantly compress the training time and the time compression rate can reach 13–50%. This indicates that the parallel training architecture proposed in this paper creates an efficient integrated detection model.

##### Comparison of different ensemble strategies

Here, we compare the detection results of a single BP neural network (None), full ensemble (Ensemble-all), by selecting a certain percentage of base classifiers with better performance for ensemble (Ensemble-30%, Ensemble-50%, Ensemble-70%), and the selective ensemble method based on adaptive clustering (SEAD-PL) proposed in this paper. The experiments are performed with the same pre-data stratified sampling and subsequent parallel training strategy, and the detection results on each dataset are listed in Table 8.

**Table 8:** Comparison of experimental results of anomaly detection under different ensemble strategies (AUC).

Method	Page blocks	Satimage-2	KDDCUP99	BATADAL	SwaT	Power	Mean
None	0.6351	0.4243	0.5766	0.7224	0.7242	0.8177	0.6500
Ensemble-30%	0.7235	0.5336	0.5887	0.7862	0.8263	0.8336	0.7153
Ensemble-50%	0.8366	0.6848	0.6475	0.8233	0.9367	0.8811	0.8017
Ensemble-70%	0.8608	0.6239	0.6729	0.8418	0.9108	0.8142	0.7874
Ensemble-all	0.8145	0.6009	0.7124	0.8361	0.9266	0.7862	0.7794
SEAD-PL	**0.9091**	**0.7782**	**0.8016**	**0.8933**	**0.9886**	**0.9506**	**0.8869**

Significant values are in bold.

From Table 5, SEAD-PL, our selective ensemble strategy based on adaptive fuzzy clustering, obtains the best detection results on all 6/6 experimental datasets. Specifically, in terms of the average AUC values on the 6 datasets, SEAD-PL shows an improvement of 0.3369, 0.1716, 0.0862, 0.095, 0.1075, respectively, considering the other ensemble strategies, especially the non-ensemble strategies that show a significant improvement in the detection effect.

**Figure 11:**
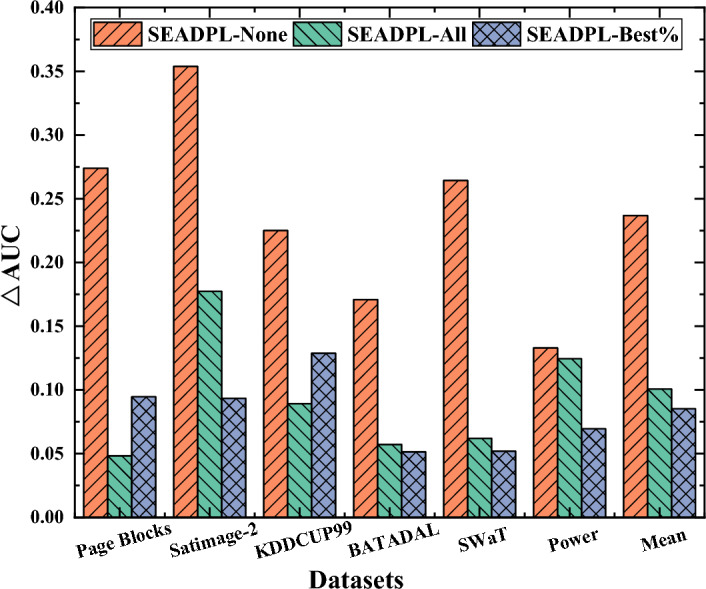
Analysis of AUC differences between SEAD-PL and different ensemble strategies.

We can also see from Table 8 that the detection effect exhibits a trend of increasing and then decreasing with the increasing ensemble scale. For example, for the Satimage-2 dataset, its AUC value increases significantly from 0.5336 to 0.6848 after the ensemble scale is increased from 30 to 50%, while it decreases to 0.6239 after the ensemble scale is further increased to 70% and further decreases to 0.6009 after the full ensemble. All other datasets and algorithms also reflect the above characteristics in terms of the average effect. This illustrates that when the ensemble scale reaches a certain level, further increasing the scale of the integrated model no longer enhances the detection effect but rather decreases it. This is because some base classifiers with poorer performance or lower differentiation will instead play a negative role when they are involved in the integrated model, and at the same time, an unreasonable increase in the ensemble scale will also elevate the temporal and spatial cost of the algorithm. This further illustrates that the selective ensemble strategy proposed in this paper has obvious advantages in determining the optimal ensemble size. Figure 11 compares and analyzes the AUC results of SEAD-PL and several comparison algorithms. The AUC values of the comparison algorithms are selected from the optimal AUC results in Table 8. As can be seen in Fig. 11, SEAD-PL exhibits better performance over the other algorithms on 6/6 experimental datasets. Moreover, the average AUC improvement of SEAD-PL ranges between 5.15 and 12.87%.

The above phenomenon is explained as follows: the comparison algorithms adopt the strategy of selective ensemble; however, the ensemble of their base classifiers pays too much attention to the individual performance of the base classifiers and insufficient attention to the group differences between independent base classifiers, leading to the introduction of too many similar detection models in the integrated model. Thus, some models with relatively poor performance rather reduce the overall detection performance. The SEAD-PL method proposed in this paper is based on a selective ensemble strategy of adaptive clustering, which considers both individual accuracy and group differences, balances the advantages and disadvantages between different base classifiers, and thus obtains better detection results.

Furthermore, the selective ensemble strategy performs better on experimental datasets with higher anomaly ratios (e.g., the Satimage-2 dataset), but this effect is not obvious on datasets with lower anomaly ratios (e.g., the Power dataset). In summary, our selective ensemble strategy based on adaptive clustering proposed in this paper has better detection performance improvement compared to other ensemble strategy algorithms.

#### Effect of the number of parallel nodes on the experimental results

The most important parameter that influences the performance of our SEAD-PL method is the setting of the number of parallel nodes. On the one hand, this parameter determines the size of the data available to each node, which in turn affects the training effectiveness of the base classifiers and the training time cost. On the other hand, the number of well-trained selective learners at these nodes provide a candidate subset for the subsequent selective ensemble. Theoretically, the larger the size of the candidate subset, the greater the variability of the base classifiers in the candidate subset and the better the performance of the selected integrated detection model; however, the larger the size of the parallel nodes, the fewer base classifiers will obtain the effective training data, and the training effect of the individuals will be affected, which in turn will lead to an accuracy reduction of the base classifiers in the candidate subset. Therefore, the setting of the number of parallel nodes is highly important. This setting also affects the time cost of training, which has been experimentally analyzed in the training pattern comparison in "Results of truncation experiments" section and will not be repeated here. Instead, we focus on the effect of the number of parallel nodes on the training effect of the base classifier and the effect on the detection effect of the integrated model.

**Figure 12:**
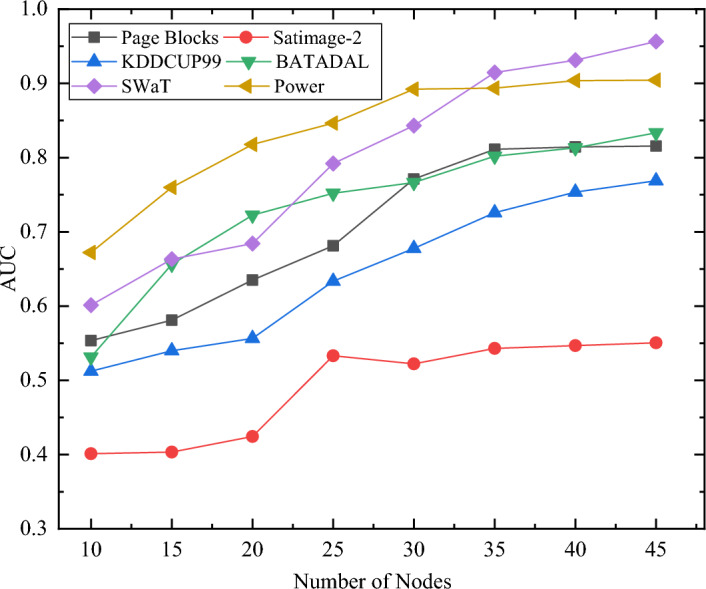
Comparison of the base classifier training effect for different numbers of parallel nodes.

Firstly, the training results of the base classifiers with different numbers of parallel nodes are shown in Fig. 12. Among them, we choose the average detection results of the base classifiers trained in all parallel nodes on the validation set for comparison.

As can be seen from the figure, the average training effect of the base classifiers increases with the growing number of parallel nodes because after grouping the data, each base classifier learns the features more adequately for a small amount of data. However, when the parallel size of one row is reached, this growth obviously stagnates since by further increasing the number of parallel nodes, the base classifiers get too little information about the samples and the features will not be sufficiently learned, thus the learning effect will be inferior. Therefore, based on the above experimental results, we can set a more reasonable number of parallel nodes to obtain a base classifier with improved individual accuracy.

**Figure 13:**
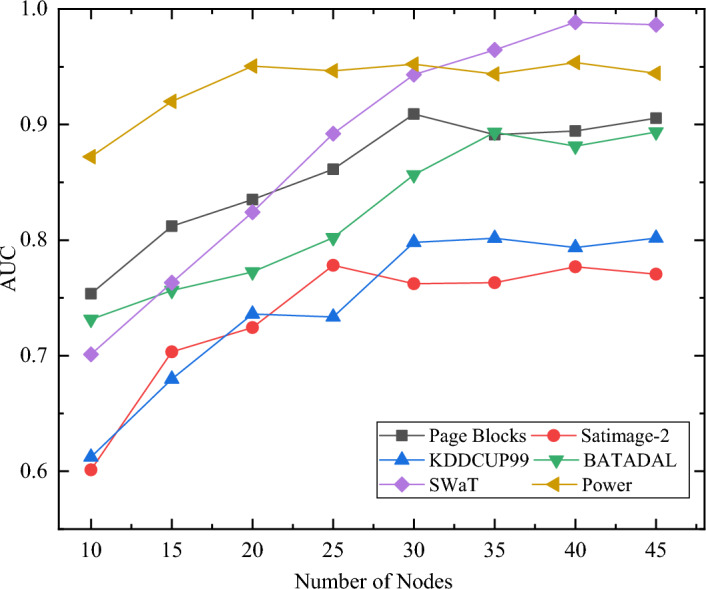
Comparison of the training effect of integrated model with different numbers of parallel nodes.

Furthermore, we test the detection effect of the integrated model on the validation set using a selective ensemble strategy with different numbers of parallel nodes. The results are shown in Fig. 13.

As can be seen from the figure, the detection effect of the integrated model improves with the increasing number of parallel nodes. This is because with more parallel nodes, the integrated model obtains a larger number of candidate subsets and the selective ensemble strategy is able to filter from a greater number of base classifiers with differentiation to improve the overall detection effect. However, when a parallel size of one row is reached, growth clearly stagnates and shows a fluctuating downward trend. As the number of parallel nodes is further increased, the base classifiers get too little sample information, the feature learning becomes insufficient, and the performance of the meta-learning effect decreases significantly (e.g., Fig. 12). Although selective ensemble can obtain more differentiated base classifiers, the decrease in individual performance also has a significant impact on the ensemble model.

Based on the experimental results above, we can set a more reasonable number of parallel nodes while taking into account the impact of the number of parallel nodes on the individual accuracy and overall variability of the base classifiers, in order to obtain a detection effect with enhanced results.

## Conclusions

Traditional anomaly detection algorithms are strongly dependent on data gauge, structure and features, exhibit large generalization errors when dealing with large amounts of multiple anomaly data from multiple domains, thus have limited applicability. To address these problems, this paper proposes a Selective Ensemble for Anomaly Detection based on Parallel Learning (SEAD-PL). First, a differentiated stratified sampling method is designed. A subset of samples with the same number of small samples is randomly extracted from the large sample set, Then, it is formed with small samples into multiple new training sets for base classifier training, which overcomes the problem of the insufficient ability of simple base classifiers and deals with extremely unbalanced data. Next, a distributed parallel training architecture is proposed. Based on the MapReduce computing framework, the training data are distributed to each node, and the edge processing capability of the distributed nodes is fully utilized for the training of base classifiers, significantly improving the training efficiency of integrated learning. Meanwhile, a clustering-based ensemble strategy is put forward. An inconsistency metric is implemented to characterize the differences ("distance") of the base classifiers, which in turn performs fuzzy clustering of the base classifiers, determines the size of the ensemble model, and selects the best-performing base classifiers in each cluster to participate in the ensemble. In this manner, the optimal ensemble effect is obtained, and the problem of balancing the accuracy and the differences of meta-learning selections in integrated learning are solved. Experimental results on several public datasets demonstrate that the method proposed in this paper has comprehensive advantages in terms of data processing, detection effect and training time. In future work, to improve the applicability of the proposed method, we aim to further extend it to the fields of multi-categorization anomaly detection, unsupervised/semi-supervised/weakly-supervised anomaly detection, and anomaly detection containing overlapping samples.

### Supplementary Information

Supplementary Information.

## Data Availability

All data generated or analysed during this study are included in this published article and its supplementary information files.
