# Supplementary material for: Selective ensemble method for anomaly detection based on parallel learning
Source: Sci Rep. 2024 Jan 16;14:1420. doi: 10.1038/s41598-024-51849-3 (PMC10791756; doi:10.1038/s41598-024-51849-3)
Supplement: Supplementary file 1 — Supplementary Information. [file 41598_2024_51849_MOESM1_ESM.docx]

1.Page Blocks:https://www.dbs.ifi.lmu.de/research/outlier-evaluation/DAMI/semantic/PageBlocks/

2.Satimage-2:https://odds.cs.stonybrook.edu/satimage-2-dataset/

3.KDDCUP99:http://kdd.ics.uci.edu/databases/kddcup99/kddcup99.html

（https://github.com/Senwei-Huang/KDD-CUP99）

4.BATADAL:http://www.batadal.net/data.html

5.SWaT:https://itrust.sutd.edu.sg/itrust-labs-home/itrust-labs_swat/

6.Power:https://data.open-power-system-data.org/
